# In Vitro Activities of Five Antifungal Drugs Against Conidia and Sclerotic Cells of Chromoblastomycosis Agent *Fonsecaea* spp

**DOI:** 10.1111/1348-0421.70038

**Published:** 2025-12-28

**Authors:** Aline Barral Takahashi, Daniella Paternostro de Araújo Grisólia, Moises Batista da Silva, Naila Ferreira da Cruz, Sâmela Miranda da Silva, Patrícia Fagundes da Costa, Claudio Guedes Salgado

**Affiliations:** ^1^ Laboratório de Dermato‐Imunologia, Instituto de Ciências Biológicas (ICB), Universidade Federal do Pará (UFPA) Marituba Brazil

**Keywords:** antifungal susceptibility, chromoblastomycosis, conidia, Fonsecaea spp, sclerotic cells

## Abstract

Chromoblastomycosis (CBM) is a chronic skin and subcutaneous infection mainly caused by *Fonsecaea pedrosoi*, a dematiaceous fungus with various morphotypes. Characteristic sclerotic cells—globe‐shaped, multiseptated and pigmented—are found in lesions of infected individuals, though their differentiation in the host remains poorly understood. To evaluate in vitro activity of five antifungal drugs—itraconazole (ITZ), posaconazole (PCZ), voriconazole (VCZ), fluconazole (FCZ), and caspofungin (CAS)—against *Fonsecaea* spp. conidia or sclerotic cells, assessing their minimum inhibitory concentration (MIC) and minimal fungicidal concentration (MFC) and correlated the ITZ MIC with patients' clinical evolution. Forty‐three clinical isolates of *Fonsecaea spp*. and the *F. pedrosoi* strain ATCC 46428 were assessed for susceptibility to ITZ, PCZ, VCZ, FCZ, and CAS following Clinical Laboratory Standard Institute guidelines (CLSI) (document M38‐A2). MIC values were determined after 5 days of incubation at 30°C, followed by MFC determination, with geometric mean MIC (GMMIC) and MFC (GMMFC) used for comparison. PCZ was the most effective antifungal drug, with geometric mean MICs of 0.3 µg/mL (conidia) and 1 µg/mL (sclerotic), and MFCs of 2.98 and 6.72 µg/mL, respectively. Clinical follow‐up revealed that higher ITZ MIC values (0.9 µg/mL) correlated with poor patient outcomes compared to lower values in improved or cured patients. These findings highlight PCZ and VCZ as promising options for CBM treatment, especially for patients not responding to ITZ.

**Abbreviations**
ATCC: American Type Culture Collection
BBL: Becton Dickinson (Bacto Brand Laboratory)
CAS: caspofungin
CBM: chromoblastomycosis
CLSI: Clinical and Laboratory Standards Institute
FCZ: fluconazole
FDA: Food and Drug Administration
GMMFC: geometric mean minimum fungicidal concentration
GMMIC: geometric mean minimum inhibitory concentration
ITZ: itraconazole
M38‐A2: CLSI reference method for broth dilution antifungal susceptibility testing of filamentous fungi
MFC: minimal fungicidal concentration
MIC: minimum inhibitory concentration
MOPS: 3‐(N‐morpholino)propanesulfonic acid
PCZ: posaconazole
RPMI: Roswell Park Memorial Institute (culture medium)
SC: sclerotic cells
SUS: Sistema Único de Saúde (Unified Health System)
VCZ: voriconazole

## 1 Introduction

CBM represents one of the primary implantation mycoses caused by melanized fungi widely found in nature that may infect agricultural workers after transcutaneous inoculation during their daily activities. This fungus lives in the soil and in decayed materials, such as woods and leaves generally occurring in one of the limbs, where it can be localized nodular, plaque, and annular or diffuse [1, 2, 3]. The main etiological agents are *Fonsecaea pedrosoi* and *Cladophialophora carrionii*, belonging to Herpotrichiellaceae family. These agents present some peculiarities in terms of geographic distribution and ecological niches, found in humid, arid and semiarid climate areas, respectively [4].

These fungi produce different morphotypes, conidia (reproduction structures) and mycelia (filamentous forms), usually found in its saprophytic lifestyle, and sclerotic cells (synonymous with muriform or Medlar bodies), which are the parasitic forms observed in the infected tissues [5]. In principle, any of these fungal forms can generate all the others, except for the transition of sclerotic cells into conidia [6]. CBM is confirmed by the presence of sclerotic cells in the skin of animal hosts [7, 8]. Sclerotic cells are believed to be highly resistant forms, characterized by multiseptated division, a very thick wall, a brownish color, and a parasitic form characteristic of chromoblastomycosis caused by different species of dematiaceous fungi [9].

The treatment of CBM remains one of the most difficult challenges in medical mycology, as no standardized therapeutic regimen has been established [10]. In practice, itraconazole (ITZ) is the most widely used antifungal, although cure rates vary widely (15%–80%). ITZ, a triazole with broad‐spectrum activity and a favorable safety profile, is usually prescribed at 200–400 mg/day, with higher doses improving response while maintaining tolerability [11, 12, 13, 14, 15]. A pulsed regimen of 400 mg/day has also been proposed to enhance cost‐effectiveness and clinical outcomes [16]. In Brazil, where CBM is endemic, the Ministry of Health provides ITZ and lipid complex amphotericin B free of charge through the Unified Health System (SUS) [17].

Despite these advances, cure rates remain suboptimal, emphasizing the need to evaluate newer azoles such as posaconazole (PCZ) and voriconazole (VCZ), which may benefit patients refractory to standard therapy, but treatment experience is limited due to the high cost and limited availability [18, 19, 20]. This study aimed to evaluate the in vitro activities of five antifungal drugs against conidia and sclerotic cells of *Fonsecaea* spp and to correlate the minimum inhibitory concentration of ITZ with the clinical evolution of the patients.

## 2 Patients and Methods

### 2.1 Ethical Aspects

This project was submitted to and approved by the Research Ethics Committee on Human Beings of the Health Sciences Center, Faculty of Pharmacy, Federal University of Pará, under protocol n° 081/07 CEP‐ICS/UFPA.

### 2.2 Treatment of Patients With Chromoblastomycosis

Forty‐three patients were diagnosed and treated by an experienced dermatologist at the Dr. Marcello Candia Reference Unit in Sanitary Dermatology of the State of Pará (URE Marcello Candia), located in the municipality of Marituba, within the metropolitan region of Belém, capital of Pará State, in Northern Brazil (Amazon region). All patients received oral itraconazole (ITZ) at daily doses ranging from 200 to 400 mg. Treatment was initiated with 200 mg/day of ITZ, regardless of clinical presentation. Dose escalation to 400 mg/day, divided into two administrations after lunch and dinner, was implemented in cases of disease progression, characterized by the appearance of new lesions or lack of clinical improvement.

Based on clinical evolution during ITZ treatment, patients were classified into four groups: (1) CURE, when there were no more clinical signs of disease activity, such as verrucous lesions, patients were submitted to a new biopsy for histopathology, direct mycological examination, and culture. ITZ was prescribed for another 3 months of treatment, and when returning, patients were “discharged for cure”, with the guidance to attend the clinic annually, or after the appearance of new lesions; (2) IMPROVEMENT, defined by the decrease in the size of the lesions; (3) NO IMPROVEMENT, when there was no change in the lesions and (4) WORSENING, when new lesions appeared during treatment.

### 2.3 Fungal Strains and Growth Conditions

**Figure 1:**
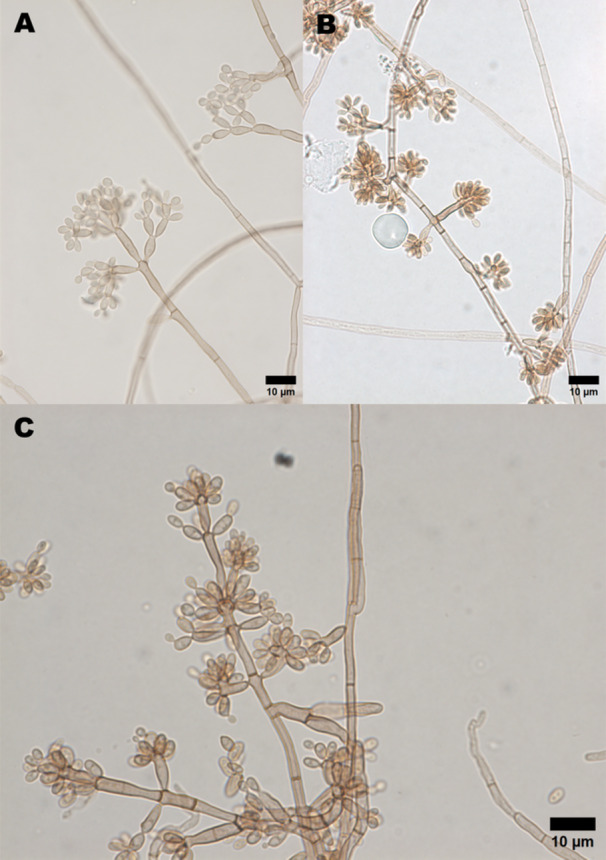
Micromorphology of *Fonsecaea* spp. isolates. Representative structures observed under slide microculture. Erect conidiophores bearing unicellular or bicellular conidia arranged in apical acropetal chains (A). Septate, melanized hyphae with conidiation (B). Detailed view of conidiophore branching and conidial arrangement (C). Scale bars = 10 µm. Morphological features were used to identify the isolates as belonging to the genus *Fonsecaea*, in accordance with established micromorphological criteria.

Forty‐three samples of CBM agents from the fungal culture collection of Dermato‐Immunology Laboratory (LDI) and *F. pedrosoi* 46428 ATCC were used. The strains were isolated in BBL Mycosel Agar (Becton Dickinson, NJ, USA) and perpetuated in potato dextrose agar (Sigma‐Aldrich, MO, USA) at 37°C for 15 days. We employed well‐established micromorphological criteria for fungal identification, including slide microculture techniques [21]. The colonies appeared black to olive in color with a yeast‐like morphology and displayed dark pigmentation on the reverse side. Micromorphological evaluation was performed to induce conidiation, which revealed structures characteristic of the genus *Fonsecaea*. These included erect conidiophores of variable length, bearing unicellular or bicellular conidia arranged apically in acropetal chains, as well as septate, melanized hyphae (Figure 1). Identification was based on morphological features described in the literature [13] allowing classification of the isolate as belonging to the genus *Fonsecaea*.

From 43 isolates, 12 were randomly selected for the induction of sclerotic cells. All selected samples successfully transformed from conidia to sclerotic cells, with an average induction period of approximately 8 days in a synthetic induction medium. The fungal cultures were harvested, suspended in 10 mL of distilled and deionized water, homogenized in a vortex for 30 s, and filtered through a nylon membrane to separate hyphae from conidia. The conidia were then collected and centrifuged at 4000 rpm for 5 min, and the pellet was resuspended in deionized water (Millipore Milli‐Q Direct 3 UV, MA, USA) to a final volume of 1 mL for counting using a Neubauer chamber (KASVI, Brazil). Then, the samples were incubated for an average of 8 days at 37°C in a chemically defined culture medium, prepared by dissolving 40 g dextrose, 19 g potassium nitrate, 40 g monobasic sodium phosphate and 9 g magnesium sulfate (Sigma‐Aldrich, MO, USA) in 1 L of deionized water, with the pH adjusted to 2.7 with HCl at a concentration of 10^4^ conidia/mL [22].

### 2.4 In Vitro Activity of Antifungal Drugs

In vitro activity of antifungal drugs to conidia and sclerotic cells was performed according to the M38‐A2 protocol of the Clinical and Laboratory Standards Institute [23]. The test concentrations of ITZ, PCZ, VCZ, and CAS (Sigma‐Aldrich, MO, USA) ranged from 0.012 to 16 µg/mL, while those of FCZ (Sigma‐Aldrich, MO, USA) ranged from 0.06 to 64 µg/mL. Quality control strain *Candida krusei* (ATCC 6258) was included in each assay run.

The samples were cultured on potato dextrose agar (Sigma‐Aldrich, MO, USA) at 37°C for 15 days and inoculum was prepared by gently scraping the surface of the colonies with a platinum handle moistened with physiological solution. The heavy particles in the cell suspension were left to stand for 3 to 5 min, after which the supernatant was transferred to a sterile tube, and the cell density was adjusted to between 0.15 and 0.17 (68%–71% transmittance) using a spectrophotometer (530 nm) (BMG LABTECH—FLUOstar Omega, Germany), corresponding approximately to a range of 10^5^–10^6^ cells/mL. This suspension was then diluted 1:50 in RPMI synthetic culture medium buffered with MOPS (Sigma‐Aldrich, MO, USA).

The MIC was determined after 5 days of incubation at 30°C and was defined as the minimum drug concentration capable of 100% inhibition of visible fungal growth compared to the control. Additionally, it was required to produce a statistically significant difference in optical density at 530 nm, measured using a microplate reader (Dynex Technologies, Chantilly, USA), when compared to groups where growth was observed.

The MFC was determined by adapting the methodology previously described by Espinel‐Ingroff [24]. Briefly, a 20 μL aliquot was subcultured from each well that showed complete inhibition (100% inhibition, or an optically clear well) from the last positive well (showing growth similar to the growth control well) and from the growth control (drug‐free medium) onto Sabouraud dextrose agar plates (Sigma‐Aldrich, MO, USA). The plates were incubated at 30°C until growth was observed in the growth control subculture (usually 120 h).

### 2.5 Statistical Analysis

Data analysis was performed using GraphPad 10 program (GraphPad Software, CA, USA) and the results were expressed as geometric mean minimum inhibitory concentration (GMMIC) and geometric mean minimum fungicidal concentration (GMMFC). To compare the GMMIC and GMMFC of conidia and sclerotic cells treated with different antifungals, we employed the Kruskal‐Wallis non‐parametric test followed by Dunn's post hoc test. For pairwise comparisons of azoles, we used paired *t*‐tests. Differences were considered statistically significant when *p* < 0.05.

## 3 Results

### 3.1 Treatment With Itraconazole

A total of 43 patients diagnosed with chromoblastomycosis (CBM) were evaluated at the Dr. Marcello Candia Reference Unit in Sanitary Dermatology (UREMC), in Marituba, Pará State, Brazil, between February 2020 and May 2024. The cohort included 42 men and one woman, with a mean age of 56.6 years (range: 34–78 years). The duration of disease prior to treatment ranged from 2 to 30 years, reflecting the chronic nature of CBM in this population.

All patients received oral itraconazole (ITZ) as monotherapy. Treatment was initiated at a daily dose of 200 mg, and in cases of disease progression or lack of clinical improvement, the dose was increased to 400 mg/day, administered in two divided doses after lunch and dinner. The clinical forms of lesions were categorized as nodular, plaque, annular, or diffuse (Table 1). Nodular lesions were the most frequent presentation, observed in 69.8% of patients, followed by plaque (23.3%), diffuse (4.7%), and annular (2.3%) forms.

**Table 1:** Clinical forms of chromoblastomycosis lesions in 43 patients treated at the Dr. Marcello Candia Reference Unit (UREMC), Marituba, Pará, Brazil. Most patients presented with the nodular form (69.8%), followed by plaque (23.3%), diffuse (4.7%), and annular (2.3%) lesions.

Clinical forms	Cases (*n* = 43)	%
Nodular	30	69.8
Plaque	10	23.3
Annular	1	2.3
Diffuse	2	4.7

Clinical outcomes were classified into four categories—cure, improvement, no improvement, and worsening (Table 2). Overall, 30 of 43 patients (69.8%) showed a favorable response to ITZ, including 25 patients (58.1%) with partial improvement and 5 (11.6%) who achieved complete clinical cure. In contrast, 13 patients (30.2%) exhibited unsatisfactory outcomes, with 8 (18.6%) showing no improvement and 5 (11.6%) experiencing disease progression.

**Table 2:** Clinical outcomes of 43 patients with chromoblastomycosis treated with itraconazole (200 mg/day). Despite long‐term therapy, complete clinical cure was achieved in only 5 (11.6%) patients. The majority, 25 (58.1%), showed partial improvement without complete resolution of lesions, while 13 (30.3%) presented unsatisfactory outcomes, including 8 (18.6%) with no improvement and 5 (11.7%) with disease progression.

Clinical outcome	Cases (*n* = 43)	%
Worsening	5	11.6
No improvement	8	18.6
Improvement	25	58.1
Cure	5	11.7

### 3.2 In Vitro Activity of Antifungal Drugs

Significant differences were observed in the GMMIC of the five evaluated drugs (*p *< 0.05). However, the concentrations required for CAS and FCZ to inhibit conidia were substantially high, prompting subsequent analyses to focus solely on the three azoles (Table 3).

**Table 3:** Antifungal susceptibility of *Fonsecaea* spp. Conidia and sclerotic Cells: Geometric means, MIC ranges, and 50th and 90th percentiles. Antifungal susceptibility profile of *Fonsecaea* spp., comparing conidia (*n* = 44) and sclerotic cells (SC; *n* = 12) for each drug tested. Results indicate higher MIC values for sclerotic cells across all antifungal agents, suggesting greater resistance compared to conidia. Among the azoles, voriconazole showed the lowest geometric mean MIC for sclerotic cells (1.12 µg/mL), followed by posaconazole (1.41 µg/mL).

Minimum inhibitory concentration (µg/mL)								
	Geometric mean		Range		50%		90%	
Antifungal drugs	Conidia (*n *= 44)	SC^a^ (*n* = 12)	Conidia (*n* = 44)	SC (*n* = 12)	Conidia (*n* = 44)	SC (*n* = 12)	Conidia (*n* = 44)	SC (*n* = 12)
Itraconazole	0.55	2.11	2–0.06	16–0.5	0.5	4	1	4
Posaconazole	0.30	1.41	1–0.12	8–1	0.25	1	0.5	1
Voriconazole	0.32	1.12	4–0.12	4–0.25	0.25	1.5	0.5	4
Caspofungin	13.7	16	> 16–4	> 16	> 16	> 16	> 16	> 16
Fluconazole	57.9	64	> 64–16	> 64	> 64	> 64	> 64	> 64

a SC, sclerotic cells.

**Figure 2:**
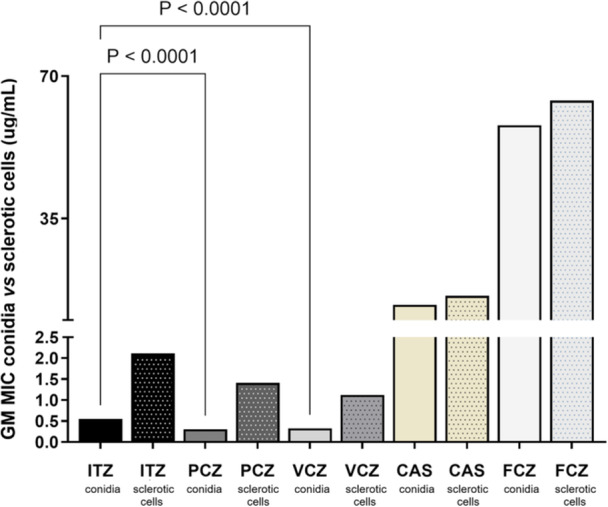
Geometric Mean Minimal Inhibitory Concentration (GMMIC) of *Fonsecaea* spp. Conidia and Sclerotic Cells for ITZ, PCZ, VCZ, CAS, and FCZ. Statistically significant differences were observed for all drugs tested in both cell types (*p* < 0.05), with PCZ and VCZ demonstrating greater efficacy against both conidia and sclerotic cells compared to ITZ (*p* < 0.0001). Sclerotic cells exhibited generally higher resistance across all antifungals tested, underscoring the increased susceptibility of conidia and sclerotic cells, especially to PCZ and VCZ.

Among these, PCZ exhibited the highest overall activity against conidia, with the lowest GMMIC (0.30 µg/mL), followed by VCZ (GMMIC = 0.32 µg/mL) and ITZ (GMMIC = 0.55 µg/mL). Significant differences were observed between ITZ and both PCZ and VCZ (*p* < 0.05), whereas no significant difference was found between PCZ and VCZ (*p* > 0.05). Regarding GMMIC against sclerotic cells, PCZ demonstrated the highest activity with the lowest GMMIC (1.41 µg/mL), followed by VCZ (GMMIC = 1.12 µg/mL) and ITZ (GMMIC = 2.11 µg/mL). Although no significant difference was found between PCZ and VCZ, both exhibited inhibitory activity at lower concentrations compared to ITZ (Figure 2).

**Figure 3:**
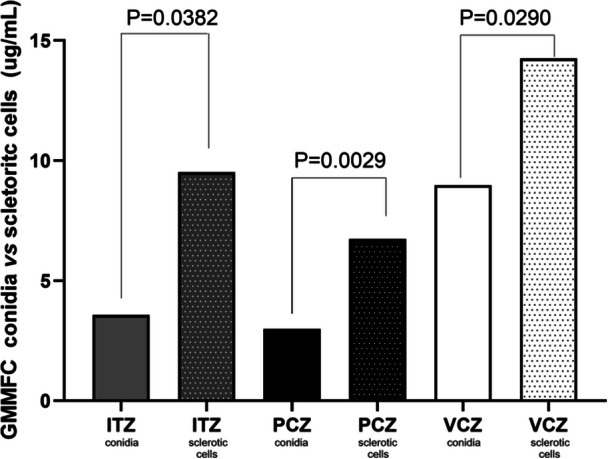
Geometric Mean Minimal Fungicidal Concentration (GMMFC) of *Fonsecaea* spp. Conidia and Sclerotic Cells for ITZ, PCZ, and VCZ. PCZ demonstrated the greatest efficacy, showing the lowest GMMFC values for both conidia and sclerotic cells. Statistically significant differences in resistance levels were observed between conidia and sclerotic cells for all drugs tested, with sclerotic cells showing higher resistance. Among the three antifungals, VCZ was the least effective.

For the MFC, PCZ proved to be the most effective drug against both conidia and sclerotic cells, with the lowest GMMFC (Table 4). Against conidia, PCZ had a GMMFC of 2.98 µg/mL, followed by ITZ with 3.56 µg/mL, and VCZ with 8.97 µg/mL. Similarly, for sclerotic cells, PCZ exhibited the highest activity, with a GMMFC of 6.72 µg/mL, followed by ITZ with 9.51 µg/mL and VCZ with 14.25 µg/mL (Figure 3).

**Table 4:** Minimal fungicidal concentrations (MFC) of *Fonsecaea* spp. Conidia and sclerotic cells: geometric means, MFC Ranges, and 50th and 90th percentiles. Fungicidal activity of five antifungal drugs against *Fonsecaea* spp., comparing the MFC for conidia and sclerotic cells (SC) based on 12 samples. The data show consistently higher MFC values for sclerotic cells, indicating increased resistance relative to conidia. Among the azoles, posaconazole exhibited the lowest geometric mean MFC for conidia (2.98 µg/mL), while it also showed relatively lower MFC for sclerotic cells (6.72 µg/mL) compared to other azoles.

Minimal fungicidal concentrations µg/mL								
	Geometric mean		Range		50%		90%	
Antifungal drugs	Conidia	SC^a^	Conidia	SC	Conidia	SC	Conidia	SC
Itraconazole	3.56	9.51	16–0.5	16–0.5	16	> 16	16	> 16
Posaconazole	2.98	6.72	8–0.12	16–1.0	8	16	16	16
Voriconazole	8.98	14.25	16–4.0	16–4.0	8	> 16	16	> 16
Caspofungin	16	16	> 16–16	> 16–16	> 16	> 16	> 16	> 16
Fluconazole	64	64	> 64–64	> 64–64	> 64	> 64	> 64	> 64

a SC, sclerotic cells.

**Figure 4:**
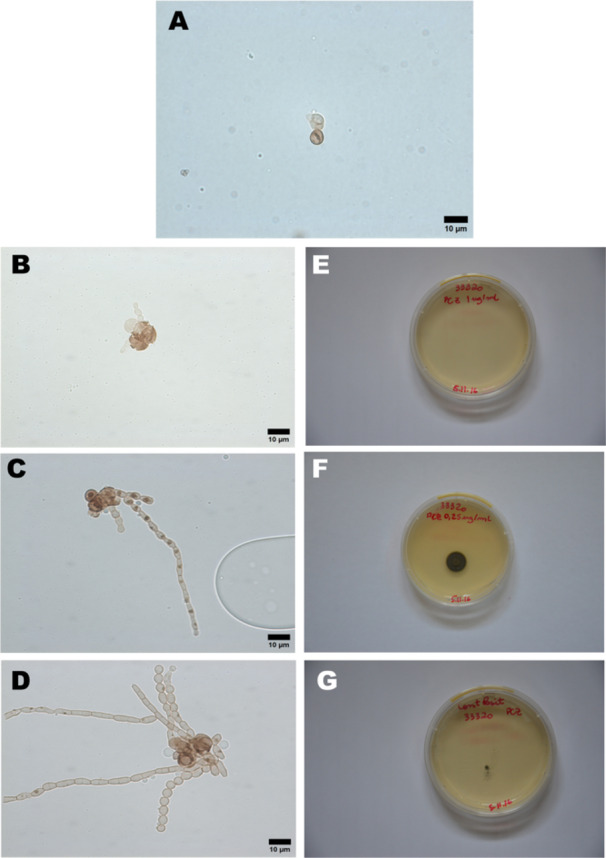
Effect of PCZ on sclerotic cells of *Fonsecaea* spp in vitro. Untreated sclerotic cells remained intact and displayed the typical brown, septated morphology (Figure 4A). A reduction in brownish pigmentation and loss of cellular morphology were observed in treated cells (Figure 4B), compared to the positive growth control (Figure 4D), indicating cellular damage. This was further confirmed by the absence of growth on solid culture medium for cells treated with PCZ at 1 µg/mL (Figure 4E). Lower concentrations (< 1 µg/mL) did not inhibit colony formation, allowing new fungal growth and hyphal differentiation from sclerotic cells (Figures 4C,F). Figure 4G shows untreated black fungal colonies in control conditions, confirming fungal growth in the absence of drug exposure.

The sclerotic cells of *Fonsecaea* spp. were exposed to drugs for 5 days (Figure 4). Optical microscopy revealed a significant fungicidal effect of PCZ at the MFC of 1 µg/mL, as seen in Figure 4B. This effect included rupture of the cell wall at multiple points, followed by cellular disorganization and potential leakage of intracellular contents. In contrast, untreated sclerotic cells remained intact and displayed normal morphology (Figure 4A).

A reduction in the brownish pigmentation of treated cells (Figure 4B) compared to the positive growth control (Figure 4D), further indicated cellular damage. This observation was confirmed by the absence of growth on solid culture medium for cells treated with PCZ at 1 µg/mL (Figure 4E). Lower concentrations (< 1 µg/mL) did not inhibit colony formation, allowing new fungal growth and hyphal differentiation from sclerotic cells (Figures 4C,F). Figure 4G shows untreated black fungal colonies under control conditions, confirming robust fungal growth in the absence of drug treatment.

### 3.3 GMMIC of ITZ Versus the Clinical Evolution of Patients

**Figure 5:**
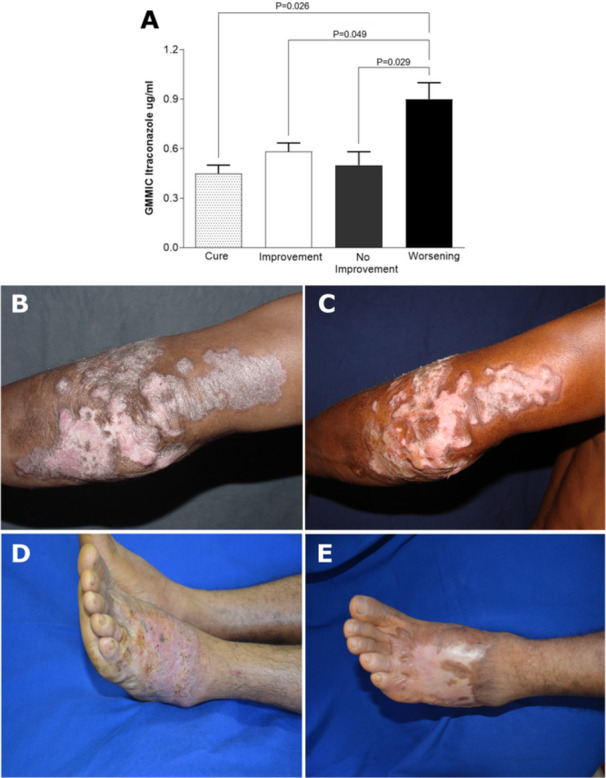
Correlation between MIC and clinical outcomes. Panel (A) presents the mean MIC values for ITZ, showing that CURE, IMPROVEMENT, and NO IMPROVEMENT groups were statistically significant in comparison to WORSENING. Panels (B) and (D) show lesions in patients with CBM before treatment, while panels (C) and (E) illustrate the condition of the lesions after treatment. Panel (C) depicts a case where the lesion persisted without improvement, leading to physical disability despite 15 years of treatment. In contrast, panel (E) shows a patient who achieved lesion regression and healing following a dose increase and 3 years of treatment, indicating a successful therapeutic outcome.

A correlation analysis was performed between the GMMIC of ITZ and the clinical evolution of patients (Figure 5). An individual analysis of each patient showed that 97,7% of the clinical isolates had a MIC ≤ 1 µg/mL. However, when the isolates were grouped according to clinical outcomes and compared based on the GMMIC of conidia (Figure 5A), the results revealed that the group classified as “worsening” had a significantly higher GMMIC value (*n* = 5; 0.9 ± 0.10 µg/mL) compared to the “cure” group (*n* = 5; 0.45 ± 0.05 µg/mL, *p* < 0.05), “improvement” group (*n* = 25; 0.58 ± 0.05 µg/mL, *p* < 0.05), and “no improvement” group (*n* = 8; 0.5 ± 0.08 µg/mL, *p* < 0.05). No statistically significant differences were observed between the “cure” group and either the “improvement” or “no improvement” groups (*p* > 0.05) (Figure 5A).

Figure 5 also presents the follow‐up of two patients. Both initially began treatment with daily doses of 200 mg and showed lesion improvement after 3 months. Subsequently, one patient experienced lesion stability without further regression or healing, prompting an increase in dosage to 400 mg/day. Despite undergoing treatment over 15‐year period, this patient showed no further improvement and eventually developed a physical disability in the affected limb (Figure 5B,C). The other patient also experienced a period of lesion stability, which required a dose increase to 400 mg/day; However, this patient showed lesion regression and complete healing after 3 years of treatment (Figure 5D,E).

## 4 Discussion

This study evaluated the in vitro activity of five antifungal drugs against *Fonsecaea* spp., comparing their efficacy on conidial and sclerotic cell forms. The results confirm that sclerotic cells—the parasitic form found in lesions—exhibit greater resistance than conidia, which are typically used in susceptibility studies [25, 26, 27, 28, 29, 30] but represent only the saprophytic form of the fungus.

Among the antifungals tested, FCZ and CAS showed minimal effectiveness against both conidia and sclerotic cells, with MICs well above therapeutic levels. As observed in previous studies, FCZ exhibited no fungicidal effect on *Cladophialophora* spp., with an MGCFM of 36.16 µg/mL [31]. CAS, an echinocandin‐class antifungal, showed high values across all evaluated parameters, thereby excluding its potential as a treatment option for CBM. This finding is consistent with prior research indicating the ineffectiveness of CAS against CBM agents [27, 32, 33]. Nevertheless, including these drugs in testing protocols can be justified, as it contributes to a comprehensive understanding of antifungal resistance and supports the development of effective therapeutic strategies against these pathogens.

PCZ showed the most potent activity against both cell types, with MIC and MFC values significantly lower than those of other azoles, such as VCZ and ITZ. Previous studies have shown that PCZ exhibited the lowest MICs against *F. pedrosoi*, followed by VCZ. Similarly, for *C. carrionii*, another CBM pathogen, VCZ demonstrated the second‐lowest MIC—both being more effective than ITZ [27, 32].

Daboit et al. identified ITZ as the most effective inhibitory agent against conidia of various pathogens including *Fonsecaea* spp., *C. carrionii*, *P. verrucosa*, *R. aquaspersa*, and *E. spinifera*, followed by VCZ [34]. Furthermore, studies by Li et al. and Feng et al. found PCZ to be the most effective against *P. verrucosa* and *Cyphellophora* isolates, followed by ITZ and VCZ, supporting our findings [29, 30].

Despite the broad use of ITZ, the higher MIC and MFC values observed for sclerotic cells in this study suggest limitations in its effectiveness for treating certain CBM cases. Clinically, patients with isolates exhibiting MIC values near 1 µg/mL for ITZ often experienced less favorable outcomes, including treatment failure. This finding underscores the need for alternative or adjunctive therapies when managing infections caused by strains with reduced ITZ sensitivity.

Although no in vitro resistance to ITZ was identified, clinical resistance—defined as a poor clinical response despite microbiological susceptibility—was observed. This phenomenon may result from host‐related factors such as age, immune status, or impaired drug absorption, rather than microbial resistance alone [23, 35, 36].

VCZ displayed intermediate effectiveness between ITZ and PCZ, with limited fungicidal action against sclerotic cells, though it may offer an alternative in cases where PCZ is inaccessible [37]. Its approval for invasive fungal infections supports its potential utility for CBM, although cost and limited availability may restrict its practical use, especially in resource‐limited settings [19, 38].

Since the mechanism of action for azole derivatives is similar—targeting lanosterol 14α‐demethylase and inhibiting ergosterol biosynthesis to the fungal cell membrane—PCZ's distinct structure, pharmacokinetics, and pharmacodynamics likely contribute to its enhanced efficacy [39].

The MFC for conidia observed in this study differed from that reported by Cardona‐Castro et al., in which ITZ was the most effective antifungal against conidia of *F. pedrosoi*, with an MFC value of 0.5 µg/mL [25], and by Vitale et al., where ITZ was also the most effective fungicidal agent, followed by VCZ, against conidia of *Cladophialophora* spp. another CBM agent [31]. In both studies, PCZ was not tested.

Our study uniquely included sclerotic cells in antifungal susceptibility testing, providing new insights into their higher resistance levels and reinforcing the importance of targeting this cellular form in severe CBM cases. The new in vitro model described here for inducing sclerotic cells may facilitate further research into their role in antifungal resistance and support the development of more effective treatments for chronic CBM. This induction period is markedly shorter than previously reported: sclerotic cell formation occurred only after 45 days in Butterfield's medium [40, 41] yeast‐like cells lacking the septation characteristic of muriform cells appeared after 30 days of culture [42], and differentiation using a chemically defined medium was achieved within 21 days [43]. These results underscore the effectiveness of the induction system employed and reinforce its applicability in experimental models investigating fungal differentiation and host–pathogen interactions.

PCZ is a systemic triazole antifungal derived from ITZ, shares the antifungal mechanism of action of other azole derivatives [37]. In Europe, approved indications include oropharyngeal candidiasis, treatment of patients' intolerant to first‐line therapies for invasive fungal disease, and salvage therapy for rare pathogens such as fusariosis, CBM, mycetoma, and coccidioidomycosis [38]. It has also been used in infections caused by other black fungi, such as *Exophiala* species. Moreover, PCZ has shown effectiveness in some cases of CBM caused by *F. pedrosoi* that were unresponsive to other therapies [20, 44].

VCZ, a newer addition to antifungal therapy, is structurally related to FCZ and exhibits a spectrum of activity comparable to ITZ. Approved by the FDA in 2002, it is indicated for invasive aspergillosis and refractory infections caused by *Scedosporium apiospermum* and *Fusarium* spp [45]. The use of PCZ and VCZ in the treatment of CBM has been reported in the literature, although clinical experience remains limited due to the high cost of these drugs [19, 27, 32].

The chemical modification of VCZ from FCZ likely contributes to its elevated MIC and MFC values against conidia and sclerotic cells of *Fonsecaea* spp. observed in our study, compared to ITZ and PCZ. Interestingly, VCZ exhibited similarly limited efficacy as FCZ against the studied samples, with the highest concentrations.

An individual analysis of each patient revealed that over 90% of isolates exhibited MIC ≤ 1 μg/mL, indicating sensitivity to ITZ. According to Revankar and Sutton, this MIC threshold is a reliable indicator of sensitivity, suggesting a favorable treatment response against melanized fungi. Furthermore, adherence to standard M38‐A2 confirmed the absence of ITZ‐resistant strains [23, 46].

Despite the lack of in vitro resistance, our study observed a rate of clinical deterioration similar to that observed in candidiasis, where 94% of strains were inhibited at ≤ 1 μg/mL of ANF, yet 60% to 70% of patients experienced therapeutic failure [47, 48]. While clinical isolates of *Fonsecaea* spp. did not demonstrate microbiological resistance, the lack of improvement in some patients suggests the presence of clinical resistance—defined as persistent or progressive infection despite appropriate antifungal therapy. Contributing factors may include antifungal pharmacokinetics, drug interactions, patient age, immune status, disease severity, and treatment adherence [35, 36].

Nevertheless, the GMMIC value remains a critical factor in disease progression. Notably, patients who experienced clinical worsening had a significantly higher mean GMMIC of 0.9 μg/mL compared to those achieving cure, improvement, or no change. These findings underscores the complex relationship between antifungal susceptibility, microbiological and clinical resistance, and their implications for individualized therapeutic strategies in managing *Fonsecaea* spp. infections.

Physicians must adhere to specific criteria to evaluate the therapeutic response in CBM patients and to determine the appropriate time to discontinue treatment. Reported cure rates with ITZ range from 15% to 80%, and evaluation criteria include clinical, mycological, and histopathological parameters [11, 12, 14, 15]. Initial ITZ dosages, typically between 200 and 400 mg/day, may vary based on physician's discretion. Treatment duration is often prolonged, with most patients showing improvement within 3 to 10 months.

A complete therapeutic response requires lesion resolution and healing, along with the disappearance of pruritus and local pain. Follow‐up should continue for at least 2 years without recurrence. The absence of fungal elements should be confirmed through direct mycological examination and culture. Histological sections should show no sclerotic cells or microabscesses; instead, granulomatous dermal infiltrates are usually replaced by chronic inflammation, dense fibrosis, and epidermal atrophy [11, 14, 49].

## 5 Conclusions

Among the five antifungal drugs evaluated, CAS and FCZ exhibited GMMICs against *Fonsecaea* spp. conidia that were 14 to 58 times higher than the CLSI's standard threshold of 1 µg/mL for sensitivity, suggesting limited effectiveness of these agents for the treatment of CBM. In contrast, ITZ, VCZ, and PCZ showed GMMICs below 1 µg/mL, with PCZ showing the most potent activity. Notably, strains with MICs approaching 1 µg/mL for ITZ were often associated with poor clinical outcomes, highlighting the need for alternative therapies in cases of reduced susceptibility.

When MIC testing was performed on sclerotic cells instead of conidia, the GMMIC values increased approximately threefold, underscoring the greater antifungal resistance of sclerotic cells. Among the drugs tested, PCZ exhibited the lowest MFC values against sclerotic cells, supporting its potential as an effective treatment option for patients unresponsive to ITZ.

These findings emphasize the importance of including sclerotic cells in antifungal susceptibility testing for CBM to better predict clinical outcomes. Our data suggests that antifungal susceptibility profiles incorporating both conidial and sclerotic cell forms, potentially through the routine use of antifungigram, could enhance treatment planning and improve patient management in CBM.

## Author Contributions

**Aline Barral Takahashi:** conceptualization, methodology, data curation, formal analysis, investigation, writing – original draft, writing – review and editing. **Daniella Paternostro de Araújo Grisólia:** conceptualization, methodology, formal analysis, investigation, writing – original draft. **Moisés Batista da Silva:** conceptualization, formal analysis, investigation. **Naila Ferreira da Cruz:** conceptualization, formal analysis, investigation. **Sâmela Miranda da Silva:** formal analysis, investigation. **Patrícia Fagundes da Costa:** conceptualization, methodology, data curation, formal analysis, investigation, writing – original draft, writing – review and editing. **Claudio Guedes Salgado:** conceptualization, methodology, data curation, formal analysis, funding acquisition, project administration and resources, supervision, writing – review and editing.

## Conflicts of Interest

The authors declare no conflicts of interest.

## Data Availability

The data that support the findings of this study are available from the corresponding author upon reasonable request.
